# Long-Term Risk of Pneumonia Among Gastric Cancer Survivors: A Nationwide Population-Based Cohort Study

**DOI:** 10.3390/cancers17223688

**Published:** 2025-11-18

**Authors:** Kyeong Min Han, Ho Suk Kang, Joo-Hee Kim, Hyo Geun Choi, Dae Myoung Yoo, Nan Young Kim, Ha Young Park, Mi Jung Kwon

**Affiliations:** 1 Hallym Data Science Laboratory, Hallym University College of Medicine, Anyang 14068, Republic of Korea; km.han@hallym.ac.kr (K.M.H.); ydm@hallym.ac.kr (D.M.Y.); 2 Division of Gastroenterology, Department of Internal Medicine, Hallym University Sacred Heart Hospital, Hallym University College of Medicine, Anyang 14068, Republic of Korea; hskang76@hallym.or.kr; 3 Division of Pulmonary, Allergy, and Critical Care Medicine, Department of Internal Medicine, Hallym University Sacred Heart Hospital, Hallym University College of Medicine, Anyang 14068, Republic of Korea; luxjhee@hallym.or.kr; 4 Suseo Seoul E.N.T. Clinic, 10, Bamgogae-ro 1-gil, Gangnam-gu, Seoul 06349, Republic of Korea; mdanalytics@naver.com; 5 Hallym Institute of Translational Genomics and Bioinformatics, Hallym University Medical Center, Anyang 14068, Republic of Korea; honeyny@hallym.or.kr; 6 Department of Pathology, Busan Paik Hospital, Inje University College of Medicine, Busan 47392, Republic of Korea; hy08.park@gmail.com; 7 Department of Pathology, Hallym University Sacred Heart Hospital, Hallym University College of Medicine, Anyang 14068, Republic of Korea

**Keywords:** gastric cancer, pneumonia, follow-up study, big data analysis

## Abstract

Background/Objectives: Gastric cancer (GC) remains a major global health burden, but its long-term association with pneumonia risk has not been comprehensively investigated. This study aimed to evaluate the long-term risk of pneumonia among GC survivors using a nationwide Korean cohort, focusing on chronic post-cancer susceptibility rather than perioperative or treatment-related complications. Methods: We conducted a nationwide, population-based cohort study using the Korean National Health Insurance Service database, including 9212 GC patients and 36,848 age-, sex-, income-, and region-matched controls (1:4 ratio). Participants were followed for up to 17 years. Propensity score overlap weighting was applied to minimize confounding, achieving exact covariate balance and optimal precision, with standardized differences used to confirm balance. Adjusted hazard ratios (aHRs) and 95% confidence intervals (CIs) for pneumonia were estimated via Cox proportional hazards models. Results: During follow-up from 2002–2003 through 2019 (maximum 17 years), GC showed a significant relationship with increased risk of pneumonia (aHR 1.06; 95% CI: 1.01–1.11; *p* = 0.014). Subgroup analyses revealed higher risks among men, socioeconomically disadvantaged individuals, urban residents, and unexpectedly, patients without comorbidities. Conclusions: This large nationwide cohort study demonstrated that GC may be linked to a slightly elevated long-term risk of pneumonia, varying across demographic and clinical subgroups. These findings underscore the need for continued respiratory health monitoring in GC survivors while acknowledging that the observed association may be influenced by underlying comorbidities and survivorship factors.

## Simple Summary

Gastric cancer (GC) patients are increasingly surviving longer due to improved screening and treatment. However, survivors may remain vulnerable to respiratory complications such as pneumonia. Using a large, nationwide cohort from Korea, we investigated the long-term risk of pneumonia following GC diagnosis. We found that GC patients had a modest but significant elevation in pneumonia risk, particularly among men, socioeconomically disadvantaged individuals, urban residents, and patients without comorbidities. These findings may emphasize the necessity of ongoing pneumonia monitoring and preventive care in GC survivorship.

## 1. Introduction

Worldwide, gastric cancer (GC) is among the most prevalent malignancies, ranking fifth in incidence and fourth in cancer-related mortality, contributing substantially to global morbidity and mortality [1]. Although its overall incidence has declined in recent decades [2], GC remains highly prevalent in East Asian countries such as Korea, Japan, and China, where dietary habits and high rates of *Helicobacter pylori* infection sustain its disease burden [3,4]. Country-specific estimates show that incidence rates per 100,000 are among the highest globally: 65.9 in Korean men, 65.8 in Japanese men, and 34.1 in Chinese men [5]. According to the Korean Central Cancer Registry, GC accounts for approximately 10% of all newly diagnosed cancers in Korea, with age-standardized incidence rates of 38.9 per 100,000 men and 17.5 per 100,000 women in 2021 [6]. Although the disease predominantly affects men, GC remains a significant health burden among Korean women, ranking as the fifth most common malignancy and the fourth leading cause of cancer-related death among women [6]. The implementation of national screening programs in Korea has led to an approximately 21% reduction in GC mortality between 2000 and 2020, contributing to improved 5-year survival compared with other regions [7]. Nonetheless, the rising incidence of GC among individuals younger than 50 years highlights the need for continued vigilance and comprehensive management strategies [2].

Pneumonia, a representative form of acute lower respiratory tract infection, is a leading cause of death worldwide, and in Korea, it was ranked as the third leading cause of death in 2019 [8,9]. In addition to its acute mortality risk, pneumonia frequently recurs, imposing substantial healthcare and socioeconomic burdens [10]. Cancer patients are at increased risk of bacterial infections due to surgical complications, chemotherapy-induced neutropenia, and the frequent use of antibiotics during cancer treatment [11,12]. Among these, pneumonia represents a major complication with substantial morbidity and mortality in this population [13]. For example, in patients with oral cancer, the incidence of pneumonia after tracheostomy was 19.71%, with higher risks observed in males and in those with prolonged tracheostomy duration [14]. Pneumonia has also been reported as the most common respiratory infection among patients with head and neck cancer and lung cancer [15]. Previous studies in various malignancies—such as lung, head and neck, hematologic, and breast cancers—have reported a persistently elevated risk of bacterial and respiratory infections long after completion of cancer treatment [16,17]. For instance, pneumonia has emerged as a leading cause of non-cancer mortality among cancer survivors, driven by immune dysregulation, malnutrition, and treatment-induced organ dysfunction [17,18]. Large population-based analyses have also demonstrated heightened susceptibility to pneumonia and sepsis in post-treatment cancer cohorts, particularly within the first 5–10 years of survivorship [13,18,19].

Beyond major surgery, the risk of pneumonia is also present in the diagnostic and therapeutic endoscopic management of GC. During upper endoscopy and particularly in endoscopic mucosal resection or endoscopic submucosal dissection, aspiration events may occur due to sedation, impairment of airway reflexes, and gastric fluid regurgitation, leading to aspiration pneumonia [20,21]. Although the incidence of pneumonia after gastric endoscopic resection was relatively low (0.62%), risk was significantly increased in older patients, smokers, and those with prolonged hemostasis time [21]. Importantly, aspiration pneumonia may develop after therapeutic endoscopy even in the absence of overt respiratory symptoms, underscoring the need for careful monitoring [20,21]. Thus, pneumonia risk extends beyond surgical settings to include endoscopic procedures, highlighting the importance of broader investigation across the continuum of GC management.

Patients with GC may be particularly vulnerable to pneumonia through multiple mechanisms [21,22]. GC itself or gastrectomy can alter the gut microbiota, which has been implicated in pulmonary disease via the gut–lung axis [23]. Postgastrectomy weight loss and malabsorption may further impair nutritional status and immune function, predisposing patients to infection [24,25,26]. Chemotherapy can also heighten pneumonia risk by inducing leukopenia and mucosal injury, weakening host defenses [27,28]. In addition, local pharyngeal anesthesia used during endoscopic procedures may suppress the gag reflex and impair upper esophageal sphincter function, thereby facilitating aspiration [20]. Aspiration pneumonia is especially relevant in cancer patients, given that swallowing dysfunction, impaired cough reflex, and anatomical changes following surgery or radiotherapy further increase aspiration risk [20,21,29].

Long-term follow-up studies have shown that cancer patients remain at elevated risk of pneumonia for years after diagnosis [30]. This persistent vulnerability has been attributed to cancer-related immune suppression, repeated healthcare exposures, and postsurgical sarcopenia [17,22,31]. However, evidence specifically addressing the long-term pneumonia risk in GC remains limited, with most prior research focused on postoperative complications [22]. They reported postoperative pneumonia incidences of 4–13% in GC and identified advanced age, male sex, chronic obstructive pulmonary disease, and extensive lymph node dissection as independent risk factors [29,32,33,34].

Therefore, the present study aimed to evaluate the long-term, post-survivorship risk of pneumonia following a diagnosis of GC, independent of short-term postoperative or treatment-related complications. Unlike previous Korean and international studies that focused primarily on post-gastrectomy or peri-treatment pneumonia, our study investigates the chronic vulnerability to respiratory infection during the extended survivorship period using up to 17 years of national follow-up data.

## 2. Materials and Methods

### 2.1. Ethics

This study was approved by the Institutional Review Board of Hallym University (IRB No: 2022-10-008), and the requirement for written informed consent was waived. All procedures were conducted in accordance with the ethical guidelines and regulations of the Hallym University Ethics Committee.

### 2.2. Study Design and Participant Selection

This study utilized data from the Korean National Health Insurance Service-National Sample Cohort (NHIS-NSC), a population-based cohort established in 2002 by the NHIS in the Republic of Korea [35]. The NHIS-NSC consists of a representative 2.2% sample of the entire Korean population, selected through systematic stratified random sampling on the basis of age (14 groups), sex (2 groups), and income (41 groups) level. Participants were followed annually until 2019 unless censored due to death or emigration, with the cohort structure maintained by the annual addition of newborns. The dataset is considered reliable and comprehensive, as its representativeness has been validated and it is continuously maintained via nationwide administrative claims data from Korea’s universal health insurance system.

From a total of 1,137,861 participants and 219,673,817 medical claim records collected between 2002 and 2019, we identified 10,174 patients diagnosed with GC. The control group consisted of 1,127,687 individuals without a diagnosis of GC during the study period. We excluded participants who had been diagnosed with GC at least once before the enrollment period (*n* = 2412) to ensure inclusion of only newly diagnosed GC cases. In addition, participants with a history of malignant neoplasms of the digestive organs (ICD-10 codes C15–C26) recorded on two or more occasions (*n* = 19,654) were excluded. These exclusions minimized potential misclassification of recurrent or pre-existing GC and ensured a clear assessment of incident GC and subsequent pneumonia risk. To enhance comparability and reduce potential bias, each GC patient was matched to four control participants via 1:4 propensity score matching on the basis of age, sex, income level, and region of residence. Control participants were randomly selected to minimize selection bias.

The index date was defined as the date of the first confirmed GC diagnosis accompanied by any therapeutic intervention (surgical, endoscopic, or chemotherapeutic) recorded in the claims database. The same index date was assigned to the corresponding matched controls to ensure temporal alignment. Pneumonia events were identified only after the index date to establish a clear temporal sequence between GC diagnosis and pneumonia onset. Pneumonia that occurred before GC diagnosis or during the diagnostic work-up period was excluded to minimize the risk of reverse causality.

**Figure 1:**
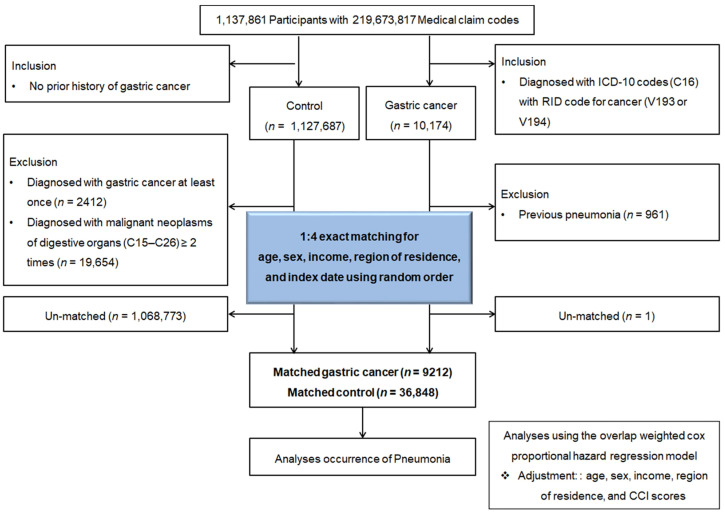
Flowchart of study population selection. From the Korean National Health Insurance Service database, 9212 patients diagnosed with gastric cancer and 36,848 matched controls were identified after applying inclusion and exclusion criteria. The final cohorts were established after excluding individuals with pre-existing pneumonia or incomplete data.

During the matching procedure, 1 GC participant and 1,068,773 control participants were excluded. Ultimately, 9212 GC patients were 1:4 matched with 36,848 control participants (Figure 1).

### 2.3. Definition of Gastric Cancer (Exposure)

GC participants were included if they were diagnosed with GC (ICD-10: C16) with rare intractable diseases (RID) codes for cancer (V193 and V194).

### 2.4. Definition of Pneumonia (Outcome)

Pneumonia was defined as a new diagnosis coded with ICD-10 codes J12–J18, clinically confirmed by either chest X-ray or computed tomography (CT) when such imaging was available during hospitalization or outpatient visit. This imaging criterion was used solely to enhance diagnostic accuracy and did not serve as an exclusion criterion for cohort enrollment. Previous pneumonia was defined as any pneumonia diagnosis (ICD-10: J12–J18) recorded before the index date; participants with such a history were excluded to avoid including recurrent or pre-existing infections. Only newly developed pneumonia events that occurred after cohort entry were included in the outcome analysis, and only the first episode after the index date was considered the outcome event to prevent duplication. Both inpatient and outpatient cases were included, as pneumonia diagnosis in the National Health Insurance Service database requires imaging-based confirmation regardless of care setting. Death before pneumonia diagnosis was treated as a competing risk event, and sensitivity analyses using Fine–Gray subdistribution hazard models were conducted to assess the impact of this factor.

### 2.5. Covariates

Participants younger than 20 years were excluded to ensure clinical relevance and consistency with prior epidemiologic studies of adult GC. Participants were stratified into 14 adult age groups (from 20–24 to ≥85 years) at 5-year intervals. Income was categorized into five levels, from level 1 (lowest) to level 5 (highest). Residential areas were classified into 16 administrative districts and further grouped as urban or rural. The comorbidity burden was assessed using the Charlson Comorbidity Index (CCI), which incorporates 17 predefined chronic conditions, including myocardial infarction, congestive heart failure, peripheral vascular disease, cerebrovascular disease, dementia, chronic pulmonary disease, connective tissue disease, peptic ulcer disease, mild or severe liver disease, diabetes (with or without complications), renal disease, hemiplegia or paraplegia, malignancy, metastatic solid tumor, and acquired immune deficiency syndrome (AIDS) [36]. To avoid overlap with the study outcomes, patients with GC were excluded from the CCI calculation.

### 2.6. Statistical Analyses

Propensity score overlap weighting was employed to balance covariates and improve the effective sample size. Propensity scores were estimated via multivariable logistic regression including all covariates, and overlap weights were calculated by assigning a weight of 1–propensity score to individuals in the GC group and propensity score to those in the control group [37]. This weighting, ranging from zero to one, was designed to achieve optimal covariate balance and enhance the precision of subsequent analyses.

We used standardized differences to compare baseline characteristics between the GC and control groups before and after weighting, with an absolute standardized difference less than 0.20 indicating adequate balance. Crude incidence rates were calculated as the number of pneumonia events per 1000 person-years.

To control for potential confounders and estimate hazard ratios (HRs) with 95% confidence intervals (CIs) for pneumonia risk in GC patients, we applied Cox proportional hazards regression with overlap weighting. Both crude and overlap-weighted models were assessed, incorporating adjustments for all aforementioned covariates.

To account for the potential impact of death as a competing event, we additionally performed competing-risk analyses using both cause-specific Cox proportional hazards models and Fine–Gray subdistribution hazard models. In these models, death was treated as a competing risk for pneumonia, and the results were expressed as cause-specific HRs and subdistribution HRs with 95% CIs.

To account for potential detection bias due to differences in healthcare utilization, we extracted data on the number of outpatient visits, hospital admission days, and imaging examinations (chest X-ray or CT) during the one-year pre-index period. These variables were incorporated into the propensity score model and included as covariates in multivariable Cox and competing-risk models. Additionally, to explore temporal patterns in pneumonia risk, we conducted a time-since-index analysis, dividing follow-up into the following intervals: 0–30 days, 31–90 days, 91–365 days, 1–5 years, and >5 years after the index date. HRs and 95% CIs were estimated separately for each interval using time-stratified Cox proportional hazards models. As a sensitivity analysis, we re-estimated pneumonia risk after excluding events that occurred within the first 6 months and 12 months following the GC diagnosis to minimize potential detection bias and to focus on long-term post-cancer vulnerability.

Statistical analyses were conducted using SAS software (version 9.4, SAS Institute Inc., Cary, NC, USA), with two-sided tests and a significance level set at *p* < 0.05.

## 3. Results

### 3.1. Baseline Characteristics

Table 1 summarizes the baseline characteristics of 9212 GC patients and 36,848 matched controls, selected at a 1:4 ratio according to age, sex, income level, and residential area. Following matching, all covariates achieved a standardized difference of 0.00, demonstrating excellent comparability between the two groups. Subsequent application of overlap weighting further minimized standardized differences to 0 for all covariates, confirming robust balance across the GC and control cohorts. These findings indicate that the matching and weighting approaches effectively reduced potential confounding and strengthened the validity of the analyses.

### 3.2. Relationship Between Gastric Cancer and Pneumonia

During the follow-up period of 45,685 person-years in the GC group and 235,866 person-years in the control group, 1301 (14.12%) and 4767 (12.94%) participants developed pneumonia, respectively. The absolute incidence rates were 28.9 and 26.7 per 1000 person-years in the GC and control groups, respectively. The crude incidence rate of pneumonia was higher in the GC group (28.5 per 1000 person-years) than in the control group (20.2 per 1000 person-years), resulting in an incidence rate difference of 8.30 (95% CI: 6.80–9.74). The crude HR for pneumonia in patients with GC was 1.37 (95% CI: 1.29–1.46, *p* < 0.001), which slightly attenuated but remained significant after adjustment with overlap weighting (adjusted HR 1.06, 95% CI: 1.01–1.11, *p* = 0.014) (Table 2).

**Figure 2:**
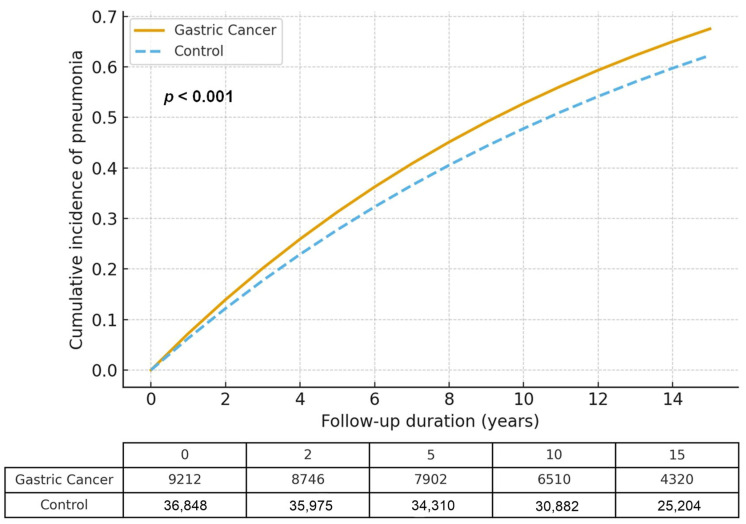
Cumulative incidence of pneumonia in gastric cancer patients and matched controls, estimated using the Fine–Gray subdistribution hazard model accounting for death as a competing event. The cumulative incidence of pneumonia remained higher in the gastric cancer group throughout the follow-up period.

In competing-risk analyses accounting for death as a competing event, the association between GC and pneumonia remained statistically significant. The cause-specific HR for pneumonia was 1.07 (95% CI: 1.02–1.12, *p* = 0.012), and the subdistribution HR estimated from the Fine–Gray model was 1.05 (95% CI: 1.01–1.10, *p* = 0.019). These results were consistent with the primary overlap-weighted Cox model (adjusted HR 1.06, 95% CI: 1.01–1.11), indicating that the elevated pneumonia risk among GC patients persisted even when accounting for death as a competing event. (*p* < 0.001; Figure 2).

**Figure 3:**
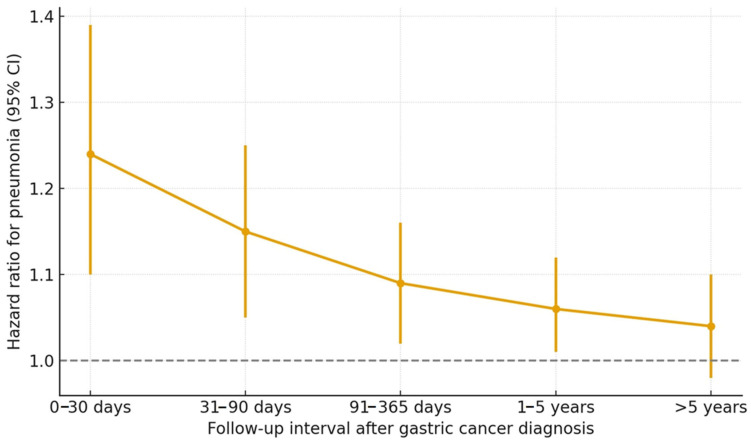
Time-since-index analysis of pneumonia risk following gastric cancer diagnosis. Hazard ratios (HRs) with 95% confidence intervals are shown for each follow-up interval. The excess risk was most prominent during the early post-diagnostic period (0–90 days) but persisted, though attenuated, during long-term follow-up.

A time-since-index analysis also showed that the excess risk was most prominent during the early post-diagnostic period (0–90 days) but persisted, though attenuated, during long-term follow-up (Figure 3). The divergence between the two groups emerged early after the index date and persisted during long-term follow-up, suggesting a sustained increased risk of pneumonia among patients with GC. In sensitivity analyses excluding early post-diagnosis pneumonia events, the association between GC and pneumonia remained statistically significant. After excluding events within 6 months, the adjusted HR was 1.05 (95% CI: 1.01–1.10; *p* = 0.021), and after excluding events within 12 months, the adjusted HR was 1.04 (95% CI: 1.00–1.09; *p* = 0.046). These findings indicate that the increased risk of pneumonia among GC survivors persists even after the early post-treatment period, supporting the hypothesis of chronic, long-term vulnerability rather than short-term peri-diagnostic effects.

### 3.3. Subgroup Analyses

Subgroup analyses using overlap weighting–adjusted models demonstrated that the risk of pneumonia was significantly higher in GC patients than in controls across several population strata. The association was particularly evident among men (adjusted HR 1.08; 95% CI: 1.02–1.14; *p* = 0.006), low-income individuals (adjusted HR 1.11; 95% CI: 1.04–1.19; *p* = 0.003), urban residents (adjusted HR 1.09; 95% CI: 1.01–1.18; *p* = 0.023), and notably, participants without comorbidities (CCI = 0; adjusted HR 1.58; 95% CI: 1.44–1.74; *p* < 0.001).

In contrast, no significant associations were observed among females, younger (<65 years) or older (≥65 years) participants, high-income or rural residents, or those with greater comorbidity burdens (CCI = 1 or ≥ 2). Interestingly, in the subgroup with a CCI ≥ 2, GC was associated with a slightly reduced risk of pneumonia (adjusted HR = 0.93; 95% CI: 0.86–1.00; *p* = 0.044).

**Figure 4:**
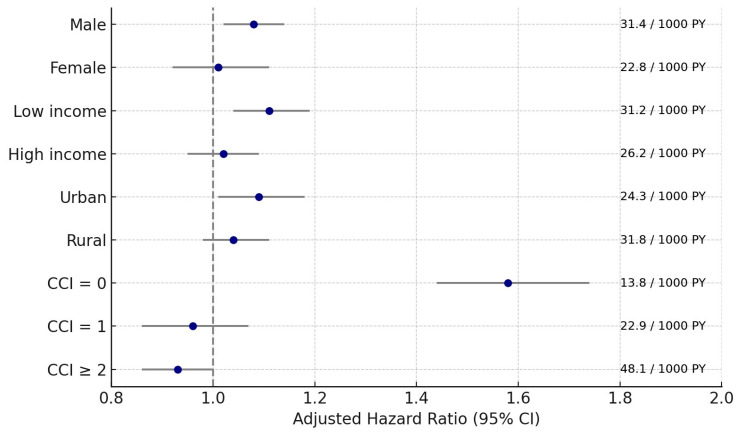
Forest plot showing subgroup-specific risks of pneumonia among gastric cancer patients compared with matched controls. Adjusted hazard ratios with 95% confidence intervals (CIs) are displayed alongside absolute risks (incidence per 1000 person-years (PY)). The risk was highest among men, socioeconomically disadvantaged individuals, urban residents, and patients without comorbidities. CCI, Charlson Comorbidity Index.

To improve interpretability, subgroup-specific adjusted HRs with 95% CIs and corresponding absolute risks (per 1000 person-years) were visualized in a forest plot (Figure 4). As shown, the elevated risk of pneumonia was most evident among men, socioeconomically disadvantaged individuals, urban residents, and patients without comorbidities.

## 4. Discussion

In this large nationwide Korean cohort, GC survivors exhibited an elevated risk of pneumonia that extended beyond the peri-treatment period. While previous Korean and international studies have primarily focused on postoperative or treatment-related pneumonia—typically occurring within the first few months after gastrectomy—our study uniquely examines chronic post-cancer susceptibility during follow-up through 2019 (maximum 17 years). This distinction is critical, as it suggests that the observed risk may result from persistent immunologic, nutritional, and physiological alterations related to GC and its sequelae rather than acute surgical or chemotherapeutic complications [17,22,31]. Importantly, the persistence of elevated pneumonia risk even after excluding early post-diagnostic events (within 6 or 12 months) reinforces the interpretation that this association reflects chronic survivorship-related vulnerability, not perioperative complications. In the crude model, GC patients exhibited a 37% higher risk of pneumonia compared with controls (HR 1.37; 95% CI: 1.29–1.46; *p* < 0.001). Although this association was attenuated after adjusting for confounders—including age, comorbidities, and socioeconomic factors—it remained statistically significant in the overlap-weighted model (adjusted HR 1.06; 95% CI: 1.01–1.11; *p* = 0.014). These findings suggest that part of the increased risk may be attributable to baseline differences between groups; however, the persistence of a significant association indicates that GC itself or its related postoperative and physiological sequelae—including anatomical alterations following gastrectomy, nutritional deficiencies, impaired swallowing function, aspiration tendency, and treatment-related immune suppression—may contribute to heightened susceptibility to pneumonia. During the follow-up period, the absolute incidence of pneumonia was 28.9 per 1000 person-years in the GC group and 26.7 per 1000 person-years among controls, representing an absolute excess of 2.2 cases per 1000 person-years. Although the relative effect was modest, this translates into a considerable number of additional pneumonia events at the population level, given the high prevalence of GC survivors in Korea.

Few studies have examined the association between GC and pneumonia in large patient populations. Our findings align with a recent meta-analysis reporting a pooled postoperative pneumonia prevalence of 11% (95% CI: 8–15%) after gastrectomy, with male sex identified as an independent risk factor (OR 3.56, 95% CI: 1.50–8.42) [38]. Notably, this meta-analysis incorporated both Asian and Western studies [38], underscoring the global relevance of this complication. Several Japanese cohorts, such as those from Shizuoka Cancer Center (*n* = 750) [39], and Kyushu University (*n* = 728, including 166 patients aged ≥75 years) [32], as well as a Chinese cohort from Fujian Medical University (*n* = 5237) [34], consistently reported postoperative pneumonia as a common and clinically meaningful complication. Korean data, although limited (*n* = 3), have documented even opportunistic infections such as *Pneumocystis jirovecii* pneumonia among GC patients undergoing chemotherapy [40]. Similarly, large-scale Western cohorts, including the REGARDS study in the United States (*n* = 29,693), have shown that cancer survivors face a 2.6-fold increased risk of infection-related morbidity and mortality [30]. Taken together, these findings highlight the global burden of pneumonia in GC patients, while East Asian studies particularly emphasize the vulnerability of elderly patients and those with impaired pulmonary function [32,33]. In Japanese cohorts, advanced age (≥75 years) emerged as an independent predictor of postoperative pneumonia, particularly in patients with concomitant sarcopenia [22,39]. In contrast, our study did not observe a clear age-related difference in pneumonia risk. Prior research has largely focused on pneumonia as a short-term postoperative complication, with cardiopulmonary comorbidities such as chronic obstructive pulmonary disease (COPD) (OR 4.72, 95% CI: 3.80–5.86), impaired respiratory function (OR 2.72, 95% CI: 1.58–4.69), hypertension (OR 2.21, 95% CI: 1.29–3.79), and prior pulmonary disease (OR 1.61, 95% CI: 1.17–2.21) identified as major risk factors [38]. These differences may reflect confounding by preexisting cardiopulmonary conditions and the heterogeneity of surgical cohorts. Unlike earlier studies restricted to postoperative populations, the present study is distinct in evaluating the long-term impact of a GC diagnosis itself—independent of treatment modality—on pneumonia risk in a nationwide population-based cohort. Aspiration pneumonia has been documented after gastric endoscopic mucosal or submucosal resection, with risk factors including advanced age and smoking, even though the overall incidence is relatively low [20,21,29]. Moreover, aspiration can occur during diagnostic upper endoscopy, particularly in patients who are elderly, immunocompromised, or undergoing the procedure under sedation, as airway reflexes are suppressed [20]. These findings suggest that pneumonia risk in GC patients may extend beyond surgical and systemic vulnerabilities to include procedure-related complications, further underscoring the need for comprehensive preventive strategies across the continuum of GC care.

Several interrelated biological and physiological mechanisms may underlie the persistent vulnerability of GC survivors to pneumonia. Following gastrectomy, nutrient malabsorption and protein–calorie deficiency are common, leading to reductions in serum albumin, vitamin B12, iron, and zinc levels—factors essential for mucosal integrity and immune cell function [41]. Chronic malnutrition and post-surgical immune suppression may compromise host defense against respiratory pathogens [42]. Moreover, sarcopenia and systemic inflammation that often develop after GC treatment can impair diaphragmatic strength and mucociliary clearance, further predisposing patients to lower respiratory tract infections [43]. Increasing evidence also points to a disruption of the gut–lung axis, whereby intestinal dysbiosis and increased microbial translocation alter systemic cytokine signaling and pulmonary immune homeostasis [44]. Reduced short-chain fatty acid production and dysregulated Toll-like receptor pathways may promote lung inflammation and infection susceptibility [45]. Collectively, these nutritional, immunologic, and microbiome-mediated pathways likely act in concert to sustain long-term pneumonia risk among GC survivors, even years after completion of cancer therapy.

Our subgroup analysis showed that the risk of pneumonia following GC was more pronounced among men, socioeconomically disadvantaged individuals, urban residents, and patients without comorbidities. The higher risk in men is consistent with previous evidence. A meta-analysis reported a postoperative pneumonia prevalence of 11% (95% CI: 8–15%), with male sex identified as an independent risk factor (OR 3.56, 95% CI: 1.50–8.42) [38]. In line with these findings, our study also demonstrated an elevated pneumonia risk in male GC patients (adjusted HR 1.08; 95% CI: 1.02–1.14). Socioeconomically disadvantaged patients may be at greater risk due to limited access to timely healthcare, nutritional deficiencies, and adverse living conditions [46], suggesting that social determinants of health may influence post-cancer outcomes. Although these observations were derived from the general population, they may plausibly explain the elevated pneumonia risk among disadvantaged GC survivors. Similarly, urban residence may reflect exposure to higher population density and environmental stressors that predispose individuals to respiratory infections. Beyond air pollution, socioeconomic and environmental disadvantages—such as crowded or inadequate housing and unequal distribution of healthcare resources—may further amplify infection risk among vulnerable populations [46]. While not specific to cancer cohorts, such findings reinforce the likelihood that environmental and social factors jointly contribute to pneumonia susceptibility in GC survivors.

Interestingly, the higher pneumonia risk observed among GC patients without comorbidities (CCI = 0) and the lower risk among those with multiple comorbidities (CCI ≥ 2) may reflect survivor bias or reverse causality rather than true protective effects [17,47]. Patients with high comorbidity burdens are more likely to die earlier from competing conditions, thereby reducing the opportunity for subsequent pneumonia diagnosis and producing an apparent inverse association [17,47]. In our sensitivity analysis excluding individuals who died within 1 year after diagnosis, the risk pattern was attenuated, supporting the interpretation that early mortality and selective survival effects contributed to these subgroup differences. Moreover, GC patients without comorbidities demonstrated an increased risk of pneumonia, a counterintuitive finding that warrants cautious interpretation. One possible explanation is detection or reporting bias: patients with preexisting comorbidities are more likely to receive closer monitoring and earlier intervention, which may reduce the likelihood of pneumonia being newly diagnosed [8,29,48]. Even after additional adjustment for pre-index healthcare utilization and time-since-index analyses, the elevated pneumonia risk persisted beyond the peri-diagnostic period. This suggests that surveillance bias alone cannot account for the association, and that residual biological or immunologic vulnerability may contribute to the long-term risk. Another consideration is statistical artifact, as the low baseline risk in this subgroup could have exaggerated the relative effect [49]. Notably, approximately 36% of cancer survivors have no comorbidities at the time of diagnosis, yet new comorbidities often rise sharply during the first year thereafter [19]. Thus, heterogeneity in immune status or treatment exposure among patients initially free of comorbidities may also contribute to their elevated pneumonia risk [23,50], underscoring the need for further investigation.

This study has several strengths, including the use of a large, representative Korean cohort, rigorous matching and weighting methods to reduce confounding, and long-term follow-up of up to 17 years. This large sample size and extended observation period enhanced the statistical power and generalizability of the findings. In addition, the application of propensity score overlap weighting helped achieve covariate balance and reduce potential confounding factors, thereby improving the comparability between GC and control groups.

However, several limitations should be acknowledged when interpreting these findings. The use of claims-based data may have introduced diagnostic misclassification, although the application of dual ICD-10 coding and imaging confirmation criteria for pneumonia likely minimized this risk. Despite the use of rigorous overlap weighting to balance baseline characteristics, residual confounding cannot be entirely excluded, as key lifestyle and clinical variables—such as smoking status, alcohol consumption, BMI, physical activity, and nutritional status—were unavailable. Moreover, detailed information on cancer stage, surgical extent, and adjuvant treatments was not accessible, precluding adjustment for treatment-related factors. Although we applied Fine–Gray competing-risk models to address mortality-related bias, we lacked access to cause-specific mortality data, which may have influenced long-term risk estimates. The subgroup findings related to socioeconomic status, urban residence, and patients without comorbidities should also be interpreted cautiously, given the potential for selection bias and unmeasured confounding.

The generalizability of this study is likewise limited, as it was conducted in a Korean population with distinct GC epidemiology and healthcare characteristics. Korea has one of the world’s highest GC incidence rates and maintains nationwide screening programs that facilitate early detection and high rates of curative surgery [1,3]. Consequently, the patterns of long-term survivorship and post-treatment complications in this setting may differ from those in Western populations, where GC typically presents at later stages and treatment approaches vary [4]. While the underlying biological mechanisms—such as immune suppression, malnutrition, systemic inflammation, and microbiome dysregulation—are likely universal [41,42,43,44], the magnitude and duration of risk may vary depending on healthcare context, ethnicity, and comorbidity structure.

## 5. Conclusions

In conclusion, this large nationwide cohort study identified a modest but significant long-term increase in pneumonia risk among GC survivors. Our findings highlight the need for comprehensive survivorship strategies focusing on infection prevention and timely follow-up care to improve long-term outcomes in this growing patient population.

## Figures and Tables

**Table 1:** General Characteristics of Participants.

Characteristics		Before PS Overlap Weighting Adjustment			After PS Overlap Weighting Adjustment		
		Gastric Cancer	Control	Standardized Difference	Gastric Cancer	Control	Standardized Difference
Age (*n*, %)				0.00			0.00
	20–24	1 (0.01)	4 (0.01)		1 (0.01)	1 (0.01)	
	25–29	18 (0.20)	72 (0.20)		12 (0.18)	12 (0.18)	
	30–34	92 (1.00)	368 (1.00)		58 (0.90)	58 (0.90)	
	35–39	197 (2.14)	788 (2.14)		133 (2.04)	133 (2.04)	
	40–44	448 (4.86)	1792 (4.86)		317 (4.86)	317 (4.86)	
	45–49	688 (7.47)	2752 (7.47)		480 (7.37)	480 (7.37)	
	50–54	961 (10.43)	3844 (10.43)		664 (10.19)	664 (10.19)	
	55–59	1145 (12.43)	4580 (12.43)		802 (12.31)	802 (12.31)	
	60–64	1345 (14.60)	5380 (14.60)		951 (14.60)	951 (14.60)	
	65–69	1334 (14.48)	5336 (14.48)		944 (14.49)	944 (14.49)	
	70–74	1307 (14.19)	5228 (14.19)		939 (14.41)	939 (14.41)	
	75–79	888 (9.64)	3552 (9.64)		642 (9.85)	642 (9.85)	
	80–84	547 (5.94)	2188 (5.94)		397 (6.09)	397 (6.09)	
	85+	238 (2.58)	952 (2.58)		173 (2.66)	173 (2.66)	
Sex (*n*, %)				0.00			0.00
	Male	6176 (67.04)	24,704 (67.04)		4366 (67.03)	4366 (67.03)	
	Female	3036 (32.96)	12,144 (32.96)		2147 (32.97)	2147 (32.97)	
Income (*n*, %)				0.00			0.00
	1 (lowest)	1761 (19.12)	7044 (19.12)		1237 (18.99)	1237 (18.99)	
	2	1159 (12.58)	4636 (12.58)		810 (12.43)	810 (12.43)	
	3	1473 (15.99)	5892 (15.99)		1040 (15.96)	1040 (15.96)	
	4	1947 (21.14)	7788 (21.14)		1365 (20.95)	1365 (20.95)	
	5 (highest)	2872 (31.18)	11,488 (31.18)		2063 (31.66)	2063 (31.66)	
Region of residence (*n*, %)				0.00			0.00
	Urban	3953 (42.91)	15,812 (42.91)		2795 (42.90)	2795 (42.90)	
	Rural	5259 (57.09)	21,036 (57.09)		3719 (57.10)	3719 (57.10)	
CCI score (Mean, SD)		2.35 (2.69)	0.81 (1.45)	0.72	1.57 (1.78)	1.57 (0.90)	0.00
Pneumonia (*n*, %)		1301 (14.12)	4767 (12.94)	0.04	860 (13.20)	1113 (17.09)	0.11

Abbreviations: PS, propensity score; CCI, Charlson Comorbidity Index; SD, standard deviation.

**Table 2:** Crude and adjusted hazard ratios (95% confidence intervals) for pneumonia in patients with gastric cancer according to age, sex, income, region, and CCI.

	Events (GC,%)	Events (Control, %)	IR per 1000 (PY)	Hazard Ratios for Pneumonia			
				Crude	*p* Value	Adjusted Model with OW ^†^	*p* Value
Overall	1301/9212 (14.12)	4767/36,848 (12.94)	28.5 vs. 20.2	1.37 (1.29–1.46)	<0.001 *	1.06 (1.01–1.11)	0.014 *
Age							
<65 years	480/4898 (9.80)	1395/19,592 (7.12)	16.5 vs. 9.8	1.66 (1.50–1.84)	<0.001 *	1.07 (0.98–1.16)	0.116
≥65 years	821/4314 (19.03)	3372/17,256 (19.54))	49.3 vs. 36.1	1.32 (1.22–1.42)	<0.001 *	1.04 (0.98–1.11)	0.156
Sex							
Male	948/6176 (15.35)	3355/24,704 (13.58)	31.4 vs. 21.6	1.42 (1.32–1.52)	<0.001 *	1.08 (1.02–1.14)	0.006 *
Female	353/3036 (11.63)	1412/12,144 (11.63)	22.8 vs. 17.6	1.26 (1.12–1.42)	<0.001 *	1.01 (0.92–1.11)	0.812
Income							
Low	648/4393 (14.75)	2298/17,572 (13.08)	31.2 vs. 20.8	1.46 (1.34–1.59)	<0.001 *	1.11 (1.04–1.19)	0.003 *
High	653/4819 (13.55)	2469/19,276 (12.81)	26.2 vs. 19.7	1.30 (1.19–1.41)	<0.001 *	1.02 (0.95–1.09)	0.552
Residence							
Urban	494/3953 (12.50)	1780/15,812 (11.26)	24.3 vs. 17.1	1.39 (1.25–1.53)	<0.001 *	1.09 (1.01–1.18)	0.023 *
Rural	807/5259 (15.35)	2987/21,036 (14.20)	31.8 vs. 22.7	1.36 (1.26–1.47)	<0.001 *	1.04 (0.98–1.11)	0.186
CCI scores		1415/23,249 (6.09)					
0	259/3270 (7.92)	1415/23,249 (6.09)	13.8 vs. 9.22	1.49 (1.30–1.70)	<0.001 *	1.58 (1.44–1.74)	<0.001 *
1	232/1795 (12.92)	1188/6492 (18.30)	22.9 vs. 28.9	0.78 (0.68–0.90)	<0.001 *	0.96 (0.86–1.07)	0.441
≥2	810/4147 (19.53)	2164/7107 (30.45)	48.1 vs. 52.5	0.88 (0.81–0.95)	0.001 *	0.93 (0.86–1.00)	0.044 *

Abbreviations: CCI, Charlson Comorbidity Index; IR, incidence rate; PY, person-year; OW, overlap weighting. * Significance at *p* < 0.05. † Adjusted for age, sex, income, and region of residence, and CCI scores.

## Data Availability

Restrictions apply to the availability of these data. The data were obtained from the Korean National Health Insurance Sharing Service (NHISS) and are available at https://nhiss.nhis.or.kr (accessed on 10 May 2025).

## References

[1] Sung H., Ferlay J., Siegel R.L., Laversanne M., Soerjomataram I., Jemal A., Bray F. (2021). Global Cancer Statistics 2020: GLOBOCAN Estimates of Incidence and Mortality Worldwide for 36 Cancers in 185 Countries. CA Cancer J. Clin..

[2] Arnold M., Park J.Y., Camargo M.C., Lunet N., Forman D., Soerjomataram I. (2020). Is gastric cancer becoming a rare disease? A global assessment of predicted incidence trends to 2035. Gut.

[3] Wong M.C.S., Huang J., Chan P.S.F., Choi P., Lao X.Q., Chan S.M., Teoh A., Liang P. (2021). Global Incidence and Mortality of Gastric Cancer, 1980–2018. JAMA Netw. Open.

[4] GBD 2017 Stomach Cancer Collaborators (2020). The global, regional, and national burden of stomach cancer in 195 countries, 1990–2017: A systematic analysis for the Global Burden of Disease study 2017. Lancet Gastroenterol. Hepatol..

[5] Jemal A., Center M.M., DeSantis C., Ward E.M. (2010). Global Patterns of Cancer Incidence and Mortality Rates and Trends. Cancer Epidemiol. Biomark. Prev..

[6] Park E.H., Jung K.W., Park N.J., Kang M.J., Yun E.H., Kim H.J., Kim J.E., Kong H.J., Im J.S., Seo H.G. (2024). Cancer Statistics in Korea: Incidence, Mortality, Survival, and Prevalence in 2021. Cancer Res. Treat..

[7] Jun J.K., Choi K.S., Lee H.Y., Suh M., Park B., Song S.H., Jung K.W., Lee C.W., Choi I.J., Park E.C. (2017). Effectiveness of the Korean National Cancer Screening Program in Reducing Gastric Cancer Mortality. Gastroenterology.

[8] Reynolds J.H., McDonald G., Alton H., Gordon S.B. (2010). Pneumonia in the immunocompetent patient. Br. J. Radiol..

[9] Wee J.H., Min C., Jung H.J., Park M.W., Park B., Choi H.G. (2022). Association between chronic rhinosinusitis and pneumonia: A longitudinal follow-up study using a national health screening cohort. Sci. Rep..

[10] Lee J.E., Kim T.H., Cho K.H., Han K.T., Park E.C. (2017). The association between number of doctors per bed and readmission of elderly patients with pneumonia in South Korea. BMC Health Serv. Res..

[11] Bhat S., Muthunatarajan S., Mulki S.S., Archana Bhat K., Kotian K.H. (2021). Bacterial Infection among Cancer Patients: Analysis of Isolates and Antibiotic Sensitivity Pattern. Int. J. Microbiol..

[12] Perez K.K., Olsen R.J., Musick W.L., Cernoch P.L., Davis J.R., Peterson L.E., Musser J.M. (2014). Integrating rapid diagnostics and antimicrobial stewardship improves outcomes in patients with antibiotic-resistant Gram-negative bacteremia. J. Infect..

[13] Kim Y.J., Lee E.S., Lee Y.S. (2019). High mortality from viral pneumonia in patients with cancer. Infect. Dis..

[14] Li L., Yuan W., Zhang S., Wang K., Ruan H. (2016). Analysis of Risk Factors for Pneumonia in 482 Patients Undergoing Oral Cancer Surgery with Tracheotomy. J. Oral. Maxillofac. Surg..

[15] Shiono S., Yoshida J., Nishimura M., Hagiwara M., Hishida T., Nitadori J.-I., Nagai K. (2007). Risk Factors of Postoperative Respiratory Infections in Lung Cancer Surgery. J. Thorac. Oncol..

[16] Chehab L., Doody D.R., Esbenshade A.J., Guilcher G.M.T., Dvorak C.C., Fisher B.T., Mueller B.A., Chow E.J., Rossoff J. (2023). A Population-Based Study of the Long-Term Risk of Infections Associated with Hospitalization in Childhood Cancer Survivors. J. Clin. Oncol..

[17] Tanaka S., Inoue M., Yamaji T., Iwasaki M., Minami T., Tsugane S., Sawada N. (2023). Increased risk of death from pneumonia among cancer survivors: A propensity score-matched cohort analysis. Cancer Med..

[18] Kawamura C., Bhaskaran K., Konishi T., Sagara Y., Bando H., Shinozaki T., Nojiri S., Adomi M., Wong A.Y.S., Tamiya N. (2025). Non-cancer risks among female breast cancer survivors: A matched cohort study in Japan. Lancet Reg. Health–West. Pac..

[19] Lee J.A., Pakpahan R., Amante D.J., Gerber B.S., Yang L. (2025). Comorbidity prevalence and incidence in cancer survivors: A longitudinal All of Us study. JNCI Cancer Spectr..

[20] Thomson A., Tye-Din J., Tonga S., Scott J., McLaren C., Pavli P., Lomas F. (2007). Aspiration in the context of upper gastrointestinal endoscopy. Can. J. Gastroenterol..

[21] Gong E.J., Kim D.H., Jung H.Y., Lim H., Ahn J.Y., Choi K.S., Lee J.H., Choi K.D., Song H.J., Lee G.H. (2014). Pneumonia after endoscopic resection for gastric neoplasm. Dig. Dis. Sci..

[22] Kamiya A., Hayashi T., Sakon R., Ishizu K., Wada T., Otsuki S., Yamagata Y., Katai H., Yoshikawa T. (2022). Long-term postoperative pneumonia in elderly patients with early gastric cancer. BMC Surg..

[23] Ma P.-J., Wang M.-M., Wang Y. (2022). Gut microbiota: A new insight into lung diseases. Biomed. Pharmacother..

[24] Davis J.L., Selby L.V., Chou J.F., Schattner M., Ilson D.H., Capanu M., Brennan M.F., Coit D.G., Strong V.E. (2016). Patterns and Predictors of Weight Loss After Gastrectomy for Cancer. Ann. Surg. Oncol..

[25] Armbrecht U., Lundell L., Lindstedt G., Stockbruegger R.W. (1988). Causes of malabsorption after total gastrectomy with Roux-en-Y reconstruction. Acta Chir. Scand..

[26] Ryan A.M., Power D.G., Daly L., Cushen S.J., Ní Bhuachalla Ē., Prado C.M. (2016). Cancer-associated malnutrition, cachexia and sarcopenia: The skeleton in the hospital closet 40 years later. Proc. Nutr. Soc..

[27] Aliberti S., Myers J.A., Peyrani P., Blasi F., Menendez R., Rossi P., Cosentini R., Lopardo G., de Vedia L., Ramirez J.A. (2008). The role of neutropenia on outcomes of cancer patients with community-acquired pneumonia. Eur. Respir. J..

[28] Rosenow E.C. (1990). Diffuse pulmonary infiltrates in the immunocompromised host. Clin. Chest Med..

[29] Kim K.H., Lee S.H., Choi C.W., Kim S.J., Ryu D.G., Choi C.I., Kim D.H., Jeon T.Y., Kim D.H., Hwang S.H. (2017). Complications and Survival Rate of Patients Over 80 Years Old Who Underwent Laparoscopic Gastrectomy for Gastric Cancer. J. Minim. Invasive Surg..

[30] Moore J.X., Akinyemiju T., Bartolucci A., Wang H.E., Waterbor J., Griffin R. (2018). A prospective study of cancer survivors and risk of sepsis within the REGARDS cohort. Cancer Epidemiol..

[31] Takiguchi H., Koyanagi K., Ozawa S., Oguma T., Asano K. (2024). Detrimental impact of late-onset pneumonia on long-term prognosis in oesophageal cancer survivors. Respir. Investig..

[32] Kimura R., Moriyama T., Ohuchida K., Shindo K., Nagai S., Ohtsuka T., Nakamura M. (2021). Risk factors for postoperative pneumonia after laparoscopic gastrectomy in patients aged 75 years and over with gastric cancer. Asian J. Endosc. Surg..

[33] Takabatake K., Sakuramoto S., Kobayashi R., Toriumi T., Ebara G., Li S., Miyawaki Y., Sato H., Yamashita K. (2024). Prognostic impact of pulmonary dysfunction in older gastric cancer patients. Sci. Rep..

[34] Tu R.H., Lin J.X., Li P., Xie J.W., Wang J.B., Lu J., Chen Q.Y., Cao L.L., Lin M., Zheng C.H. (2017). Prognostic significance of postoperative pneumonia after curative resection for patients with gastric cancer. Cancer Med..

[35] Lee J., Lee J.S., Park S.H., Shin S.A., Kim K. (2017). Cohort Profile: The National Health Insurance Service-National Sample Cohort (NHIS-NSC), South Korea. Int. J. Epidemiol..

[36] Quan H., Li B., Couris C.M., Fushimi K., Graham P., Hider P., Januel J.M., Sundararajan V. (2011). Updating and validating the Charlson comorbidity index and score for risk adjustment in hospital discharge abstracts using data from 6 countries. Am. J. Epidemiol..

[37] Li F., Thomas L.E., Li F. (2019). Addressing Extreme Propensity Scores via the Overlap Weights. Am. J. Epidemiol..

[38] Fan S., Jiang H., Xu Q., Shen J., Lin H., Yang L., Yu D., Zheng N., Chen L. (2025). Risk factors for pneumonia after radical gastrectomy for gastric cancer: A systematic review and meta-analysis. BMC Cancer.

[39] Miki Y., Makuuchi R., Tokunaga M., Tanizawa Y., Bando E., Kawamura T., Terashima M. (2016). Risk factors for postoperative pneumonia after gastrectomy for gastric cancer. Surg. Today.

[40] Yoon S.Y., Ki H.K., Kim S.Y., Cho Y.H., Lee H.G., Yoo M.W. (2012). Pneumocystis carinii pneumonia in gastric cancer patients without acquired immune deficiency syndrome: 3 cases report and literature review. J. Korean Surg. Soc..

[41] Triantafillidis J.K., Malgarinos K. (2024). Immunonutrition in Operated-on Gastric Cancer Patients: An Update. Biomedicines.

[42] Santos S.S., Costa L.A.T.J.d., Araripe T.S.d.O., Reges B.D.L.O., Ximenes H.M.d.A., Moreira A.C.d.O.M. (2025). Immunomodulatory enteral nutrition in post-surgical gastrointestinal cancer: Clinical, biochemical and nutritional impacts. Clin. Nutr. ESPEN.

[43] Fu M., Wang X., Zhou J., Wang J. (2025). Incidence and risk factors of sarcopenia in gastric cancer patients: A meta-analysis and systematic review. BMC Cancer.

[44] Indari A., Rasmin M., Syam A. (2023). Gut-Lung Axis. Respir. Sci..

[45] Zhao Y., Liu Y., Li S., Peng Z., Liu X., Chen J., Zheng X. (2021). Role of lung and gut microbiota on lung cancer pathogenesis. J. Cancer Res. Clin. Oncol..

[46] Bourgeois A., Horrill T., Mollison A., Stringer E., Lambert L.K., Stajduhar K. (2024). Barriers to cancer treatment for people experiencing socioeconomic disadvantage in high-income countries: A scoping review. BMC Health Serv. Res..

[47] Panigrahi G., Ambs S. (2021). How Comorbidities Shape Cancer Biology and Survival. Trends Cancer.

[48] Liu L., Deng W. (2023). Gastric Cancer: Innovations in Screening, Diagnosis and Treatment. J. Pers. Med..

[49] Santry H.P., Wren S.M. (2012). The role of unconscious bias in surgical safety and outcomes. Surg. Clin. N. Am..

[50] Guo J., Wang L., Han N., Yuan C., Yin Y., Wang T., Sun J., Jin P., Liu Y., Jia Z. (2024). People are an organic unity: Gut-lung axis and pneumonia. Heliyon.

